# Isolation and pathogenicity of porcine circovirus type 2 in mice from Guangxi province, China

**DOI:** 10.1186/s12985-023-02161-5

**Published:** 2023-08-29

**Authors:** Qiulin Jiao, Liuyue Yang, Xiangzu Liu, Yanwen Wen, Linxing Tian, Ping Qian, Huanchun Chen, Xiangmin Li

**Affiliations:** 1 National Key Laboratory of Agricultural Microbiology, Hubei Hongshan Laboratory, Huazhong Agricultural University, Wuhan, Hubei 430070 PR China; 2 College of Veterinary Medicine, Huazhong Agricultural University, Wuhan, Hubei 430070 PR China; 3 Key Laboratory of Preventive Veterinary Medicine in Hubei Province, the Cooperative Innovation Center for Sustainable Pig Production, Wuhan, Hubei 430070 PR China

**Keywords:** Porcine circovirus type 2, Isolation, Phylogenetic analysis, Pathogenicity

## Abstract

**Background:**

Porcine circovirus type 2 (PCV2), a member of the genus *Circovirus* and family *Circoviridae*, is a closed, small, circular, and single-stranded DNA virus, and it is a crucial swine pathogen of porcine circovirus-associated diseases (PCVADs). PCV2 was first detected in PK-15(ATCC-CCL) cells in 1974, which has caused significant economic loss to the swine industry throughout the world. And the first case of PCV2 was reported in China in 2000. At present, PCV2d is the main genotype circulating widely in China.

**Methods:**

Lymph samples were obtained from piglets with emaciation and respiratory disease in Guangxi province, China. The main pathogens were detected via PCR from lymph samples, and then PCV2-single positive samples were used to inoculate with PK-15 cells. After successive generations, the isolate was subsequently identified by polymerase chain reaction (PCR), immunofluorescence assay (IFA), Western blot (WB), and transmission electron microscopic (TEM). The full-length genome and genetic characterization of isolates were analyzed by Sanger sequencing. The TCID_50_ of the PCV2-GX-6 was determined by IFA, and the pathogenicity of PCV2 in BALB/c mice was analyzed via the mouse model.

**Results:**

The isolates were successfully isolated from clinical samples. The complete genome of PCV2-GX-4, PCV2-GX-6, PCV2-GX-7, PCV2-GX-11 and PCV2-GX-16 have been amplified, sequenced, and deposited in GenBank (accession no.: OR133747, OQ803314, OR133748, OR133749, OR133750). Homology and phylogenetic analysis with reference strains showed that the isolates belonged to the PCV2d genotype. The PCV2-GX-6 could be stably passaged more than 30 times in PK-15 cells. PCV2-GX-6 was identified by PCR, IFA, WB and TEM. The results of homology showed that PCV2-GX-6 was closely related to the reference strains PCV2-JS17-8 (GenBank accession no.: MH211363). Pathogenicity studies in mice have shown that PCV2-GX-6 can lead to growth inhibition of mice. Meanwhile PCV2-GX-6 caused the typical lesions of spleen, lung and kidney. The results of qPCR showed that PCV2 can effectively proliferate in the liver, spleen, lung, and kidney.

**Conclusion:**

PCV2-GX-6 can successfully infect BLAB/c mice, effectively proliferate in major organs, and possessed high pathogenicity. In conclusion, combined with the genotype and pathogenicity of PCV2d currently prevalent, PCV2-GX-6 can be used as a candidate vaccine strain.

**Supplementary Information:**

The online version contains supplementary material available at 10.1186/s12985-023-02161-5.

## Introduction

Porcine circovirus type 2 (PCV2) is a member of the genus *Circovirus* and family *Circoviridae*, a closed, small, circular, single-stranded DNA virus [1], and it is a crucial swine pathogen of porcine circovirus-associated diseases (PCVADs) [2]. 9 major genotypes of PCV2 have been identified based on phylogenetic classification of the complete genome and ORF2 sequences: PCV2a, PCV2b, PCV2c, PCV2d, PCV2e, PCV2f, PCV2g, PCV2h and PCV2i [3, 4]. The diversity of genotypes brought more potential dangers to the pig industry.

ORF2-targeted gene sequencing is commonly used as a gold standard for phylogeny, epidemiology, and genotyping of PCV2 strains [5]. The transformation of PCV2 genotypes changed the virulence of the virus. Previous studies determined that the shift from genotype PCV-2a to PCV-2b occurred in 2003 on a more global scale, the clinical symptoms associated with PCV2 infection are more typical [6]. Recent epidemiological studies on PCV2 revealed that PCV2d is becoming the most important epidemic strain at present [7, 8]. PCV2 caused porcine circovirus-associated diseases (PCVADs), including postweaning multisystemic wasting syndrome (PMWS), porcine dermatitis and nephropathy syndrome (PDNS), porcine respiratory disease complex (PRDC), proliferative and necrotizing pneumonia (PNP), reproductive failure, and respiratory diseases [9, 10]. The diversity of clinical symptoms also revealed that PCV2 posed a great threat and economic losses to the health of pig population and the development of the industry [11–13].

The diversity of PCV2 genotypes made the occurrence of multisystem diseases more frequent, which caused greater challenges to the prevention of diseases and the selection of vaccines. Additionally, there are relatively few reports about the pathogenesis, immune responses, and development of novel vaccines for PCV2. In this study, we investigated the genetic diversity and evolutionary relationship of isolates and reference strains. The results showed that isolates belonged to the PCV2d genotype. The infection study on mice confirmed the pathogenesis of PCV2-GX-6. Methods and strategies in experiments provided guidance for the isolation, identification, and pathogenicity study of the PCV2.

## Materials and methods

### Nucleic acid extraction and pathogen detection

In 2022, lymph samples of piglets with respiratory diseases and emaciation were collected from a large-scale pig farm in Guangxi. Appropriate tissue was homogenized and diluted with sterile PBS, freeze-thawed twice at -80℃, and centrifuged at 9,000 rpm for 10 min at 4℃ to collect the supernatant. DNA was extracted using an E.Z.N.A.® Viral DNA Kit (Omega Bio-tek, Georgia, USA) following the manufacturer’s protocol. RNA was extracted using TRIzol reagent in accordance with the manufacturer’s protocol instructions. A series of primers (Additional fle 1: Table S1) were designed to detect pathogens, including classical swine fever virus (CSFV), porcine reproductive and respiratory syndrome virus (PRRSV), porcine pseudorabies virus (PRV), porcine circovirus type 3 (PCV3), porcine circovirus type 4 (PCV4) using methods as previously described [14–18]. And the amplification of DNA/cDNA was performed using the following conditions: (i) 95 °C for 3 min, (ii) up to 32 cycles of 95 °C for 15 s, 56 °C 30 s, and 72 °C for 20 s, (iii) 72 °C for 10 min. A single positive PCV2 was determined by the above tests for virus isolation in the next step.

### Isolation and identification of PCV2

The isolates were isolated and identified on PK-15 cells, PK-15 cells were cultured in high-glucose DMEM (Dulbecco’s modified Eagle medium, HyClone, Marlborough, MA, USA) supplemented with 10% (v/v) FBS (heat-inactivated fetal bovine serum, Inner Mongolia opcel Biotechnology Co., Ltd., Inner Mongolia, China). When the cell growth density reached 70%. The supernatant of the homogenate with a single positive PCV2 was filtered and inoculated into the cells. After incubation at 37℃ and 5% CO_2_ for 2 h, the DMEM containing 2% FBS was replaced. After 72 h, the cells were frozen and thawed twice at -80℃ and centrifuged at 12,000 rpm for 10 min at 4℃. The virus was harvested and inoculated into PK-15 cells. After three consecutive passes, PCR was performed to identify the virus by PCV2-specific primers. According to the above method, a isolate was stably cultured in a PK-15 cells culture system for 33 passages and labeled PCV2-GX-6.

The immunofluorescence assay (IFA) was applied for the identification of PCV2 [19]. The virus and PK-15 cells were incubated in 24-well plates for 2 h and then replaced with DMEM containing 2% FBS for 72 h. Infected PK-15 cells were washed with PBS for 3 times and fixed with pre-cooling 100% methanol at -20℃ for 20 min, then washed with phosphate buffer containing Tween 20 (PBST) for 3 times and blocked with PBST containing 5% BSA and 0.3% Trifonx-100 at 37℃ for 1 h. After the cells were washed with PBST for 3 times, the blocked cells were incubated with PCV2 monoclonal antibody for 2 h at 37℃. Then, washing with PBST again. The infected cells were incubated with goat anti-mouse fluorescent antibody (TermoFisher, Waltham, MA, USA) at 37℃ for 1 h. The cells were washed with PBST for 4 times and incubated with DAPI at 37℃ for 15 min. After intensive washing, the cells were observed and analyzed under a fluorescence microscope (Ti-U-Nikon, Tokyo, Japan) with a video documentation system. The virus was identified by Western blot assay (WB) using a Cap monoclonal antibody of PCV2. PK-15 cells were suspended at 72 h post-infection and centrifuged at 1,000 rpm for 5 min at 4℃. Then, cells were digested by NP40 for 30 min. The isolates were separated by using 12% sodium dodecyl sulfate-polyacrylamide gel electrophoresis (SDS-PAGE) and then transferred to polyvinylidene difluoride membranes. PBST containing 5% skim milk was used to block the membrane at 4℃ for 1 h, after washing by PBST, the membrane was incubated with Cap monoclonal antibody for 2 h at 37℃, and then washed with PBST for 3 times. The membrane was incubated by the HRP-labelled goat anti-mouse antibody (ABclonal China) at 37℃ for 1 h. After intensive washing, the signal was observed using the ECL chemiluminescence system. Protein bands were analyzed by Image Lab software 4.0.1.

### Determined of TCID_50_ of PCV2-GX-6 by IFA

The PCV2-GX-6 was continuously diluted 10 times (10^− 1^-10^− 8^) with DMEM. Different concentrations of the virus were mixed with PK-15 cells, and each concentration was repeated 8 times. Normal PK-15 cells were used as the negative control group. Cells with intranuclear green fluorescence were found to be positive, and TCID_50_ of PCV2-GX-6 was calculated according to Reed-Muench methods.

### Phylogenetic analysis

Isolates were amplified by PCR using specific primers (Additional fle 1: Table S2), the purified product was connected to the pMD18-T vector (TaKaRa, Dalian, China) and transformed into DH5a, the plasmid was extracted and sequenced according to instructions, respectively. The sequences of DNA were compiled and edited using Lasergene 7.1 (DNASTAR, Madison, WI, USA). Genetic distance between the isolates and reference strains was determined by genetic evolution analysis of nucleotides and amino acids. The phylogenetic tree was constructed by the neighbor-joining method with 1000 bootstrap replicates via MEGA 7.0 (Sinauer Associates, Inc., Sunderland, MA, USA). PCV2-GX-6 Cap protein structure predicted by Swiss model(https://swissmodel.expasy.org/), mapped and compared with reference strain by Pymol (BOUNDLESS, Schrödinger, GER).

### Pathogenicity studies in mice

To study the pathogenicity of the isolates in mammals, the six-week-old female BALB/c mice were selected for infection test. Mice were purchased from the Laboratory Animal Research Center of Huazhong Agricultural University (Wuhan, China) and the test procedure was approved by the Ethical and Welfare Committee (HZAUMO-2023-0012). The copy number of the PCV2 was detected by qPCR, and PCV2-GX-4, PCV2-GX-6 and PCV2-GX-11 with higher copy number was selected. The mice were randomly divided into 4 groups, and infected with PCV2 (1 × 10^6^ TCID_50_). The control mice were infected with DMEM. Clinical symptoms and body weight of the mice were monitored for 14 days. The results showed that PCV2-GX-6 has obvious pathogenicity to mice. To further explore the pathogenicity of PCV2-GX-6. The mice were randomly divided into 2 groups with 10 mice. And the mice were infected with PCV2-GX-6 (1 × 10^6^ TCID_50_). The negative control was infected with DMEM. The lactating female mice were intraperitoneally anesthetized with ketamine (87 mg/kg) and xylazine (13 mg/kg). Ten minutes after anesthesia, 200 µL of inoculum was injected into the muscle, abdominal cavity, and back, respectively. The weight of mice was monitored from 1 dpi to 14 dpi. And the heart, liver, spleen, lung, kidney, and brain were collected from 5 mice (humanely euthanized by injecting an overdose of intraperitoneal sodium pentobarbital) randomly selected from each group at 7dpi after infection. Part of the tissue samples were fixed with 4% paraformaldehyde solution (Biosharp life sciences, Beijing Labgic Technology Co., Ltd., Beijing, China) for histopathological analysis, and the others were stored at -80℃ to detect viral load and distribution of the virus in different organs by qPCR.

### Viral load analysis

To detect the viral load of PCV2 in infected mice. The pEASY-Blunt-PCV2 plasmid was used as the standard plasmid to draw a standard curve, and the copy number of PCV2 was calculated using the standard curve. Various tissues of the infected mice were homogenized and diluted with sterile PBS, DNA was extracted using E.Z.N.A.® Viral DNA Kit (Omega Bio-tek, Georgia, USA) in accordance with the manufacturer’s protocol. Real-time quantitative PCR (qPCR) was carried out in the ViiA™ 7 Real-Time PCR System (Applied Biosytems, Grand Island, NY, USA) system with Go Taq® G2 Hot Start Polymerase (Promega (Beijing) Biotech Co., Ltd, Beijing, China.), according to the manufacturer’s instructions.

### Histopathological analysis

Haematoxylin and eosin (HE) staining analysis was performed to observe the pathological changes in mouse tissues. Tissues fixed in 10% neutral buffered formalin were embedded in paraffin and cut into serial sections using a manual rotary microtome (HistoCore BIOCUT, Leica Biosystems, Shanghai, China).

### Statistical analysis

The statistical analyses of pathogenicity studies in mice were performed using GraphPad Prism 8.3.0 software. Comparisons among different groups were evaluated by one-way ANOVA. Data were expressed as the mean ± the standard deviation (SD). In all cases, *p* < 0.05 was considered as statistically significant difference.

## Results

### Detection, isolation, and identification of pathogens

**Fig. 1:**
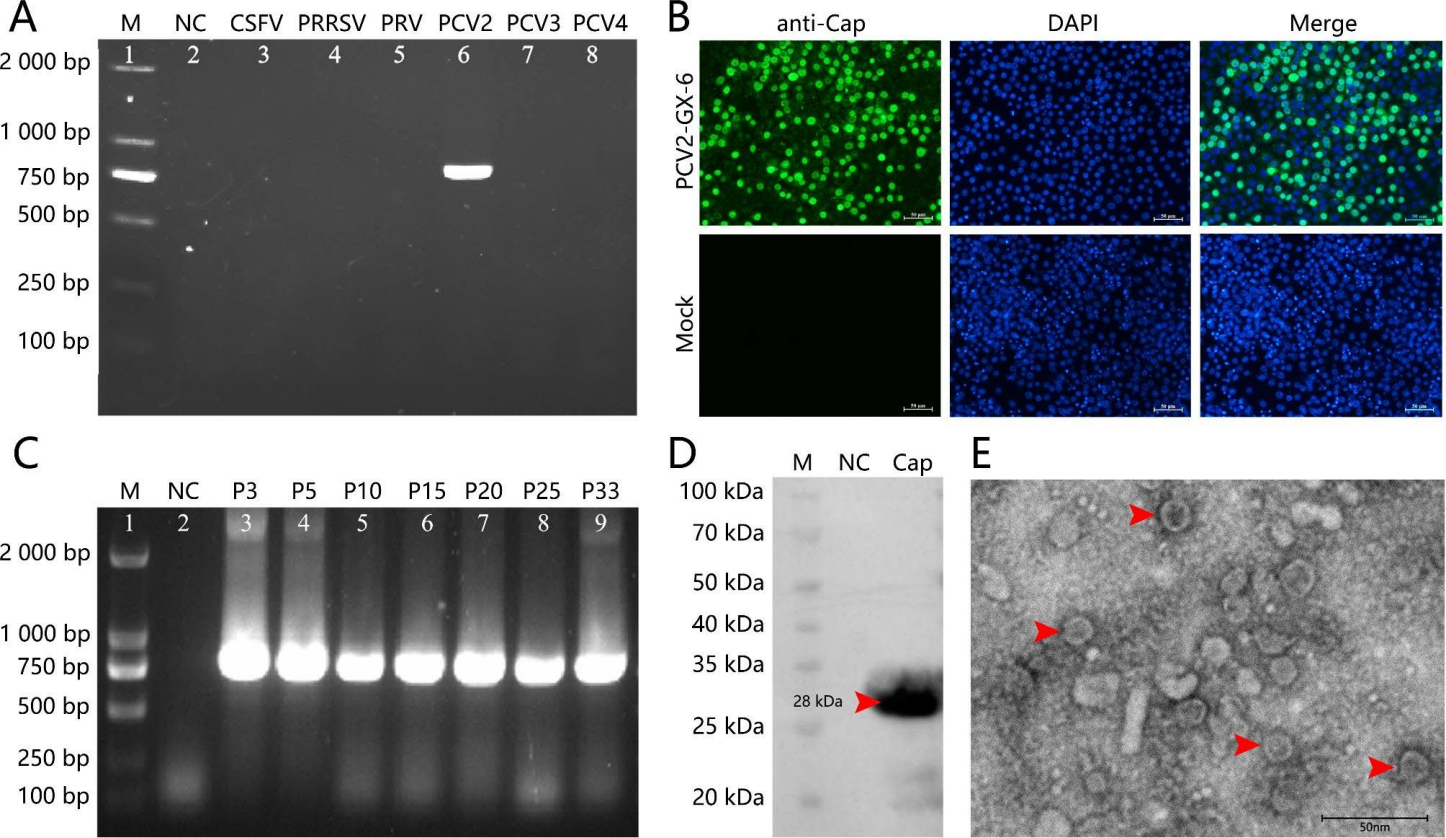
Identification of PCV2-GX-6 strain. (**A**) PCR detection of PCV2 in the swine lymph. PCR amplification identified the causative agent using CSFV, PRRSV, PRV, PCV3, and PCV4 specific primers, ddH_2_O was used as a negative control (Lane 2). (**B**) Immunofluorescence assay (IFA) of PK-15 cells infected with PCV2-GX-6 strain at 72 h post-infection. The viral protein was detected in PK-15 cells inoculated with PCV2 Cap mAb, and mock-infected cells (PK-15) were used as the negative control. (**C**) Identification of the different generation isolates by PCR using the specific primer, ddH_2_O was used as a negative control (Lane 2). (**D**) Detection of PK-15 cells infected with PCV2-GX-6 strain by Western blot using Cap monoclonal antibody of PCV2. (E) Identification of virus by Transmission electron microscopic shows features of diameter 17nmparticle by negative stain (Bar = 50 nm).

In 2022, PCV2 was detected in piglets with clinical emaciation and respiratory disease (cough, wheezing) in Guangxi, China. In addition, PCV2 was detected as a single positive by RT-PCR and PCR (Fig. 1A). IFA results showed that the fluorescence of PCV2-GX-4, PCV2-GX-7, PCV2-GX-11 and PCV2-GX-16 in PK-15 cells was low (Additional fle 2: Fig. S1). We successfully proved PCV2-GX-6 colonization in the nucleus of PK-15 cells by IFA (Fig. 1B), and the TCID_50_ of PCV2-GX-6 was 10^-7.125^/mL according to Reed-Muench method. PCV2-GX-6 could be detected by PCR after 33 passages (Fig. 1C). The result indicated the stability of virus-infected cells. The virus was detected with bands at 28.0 kDa by Western blot using PCV2 Cap monoclonal antibody as primary antibody (Fig. 1D). TEM results revealed that the purified virus exhibited spherical particles with a diameter of approximately 17 nm (Fig. 1E). Thus, based on the above identification results, the PCV2-GX-6 was successfully isolated in this study.

### Phylogenetic analysis

**Fig. 2:**
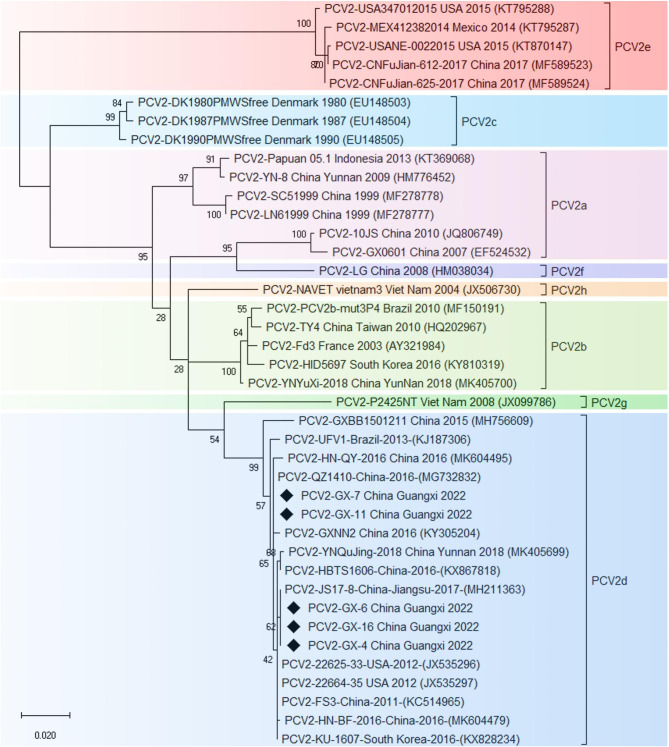
Phylogenetic analysis of Cap gene. Phylogenetic tree was constructed in MEGA version 7.0 using the neighbor-joining method with the Maximum Composite Likelihood model with 1000 bootstrap replicates. The isolates were marked “◆”

**Fig. 3:**
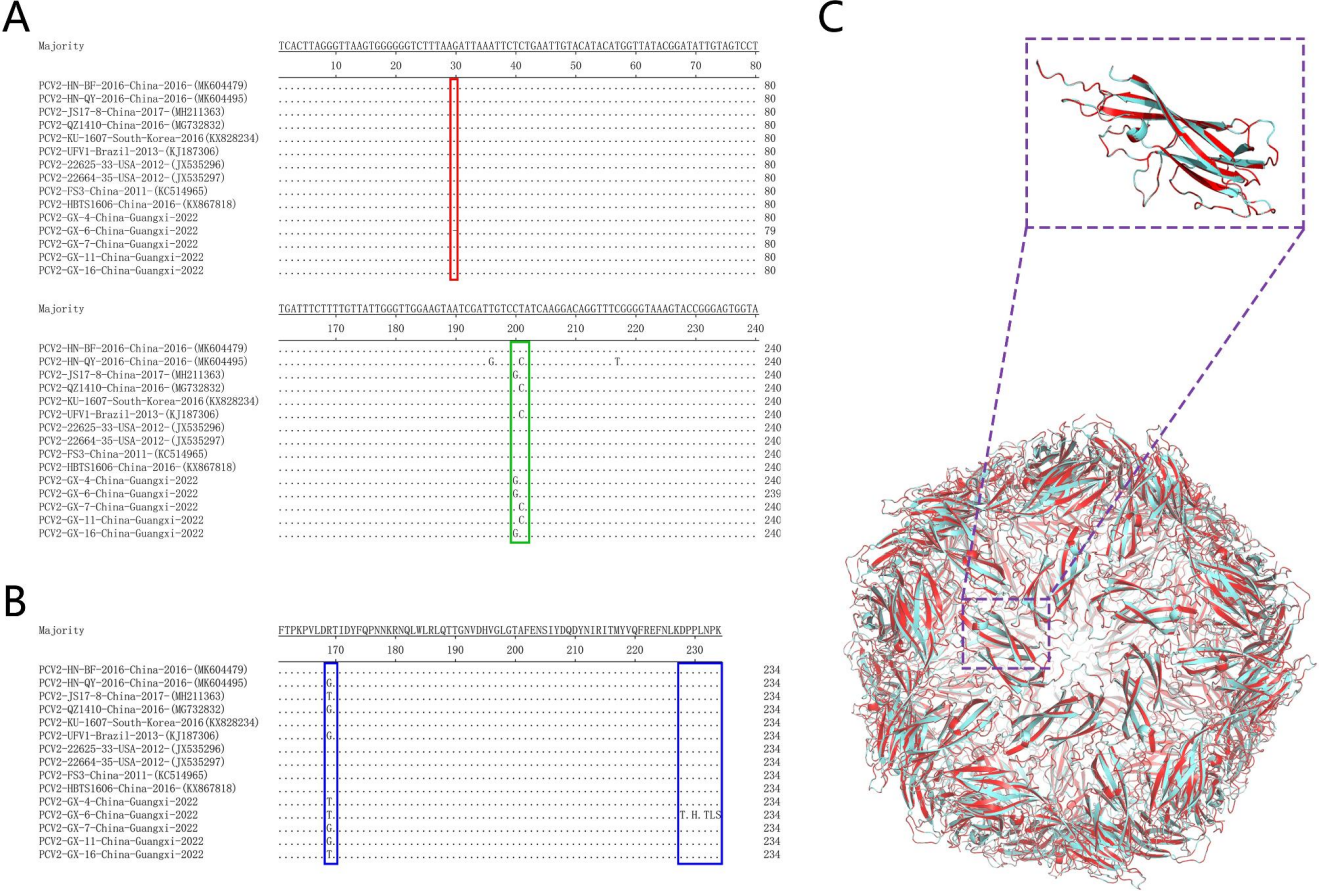
Homology analysis of Cap nucleotide and amino acid sequences of the isolates and reference strains. (**A**) Nucleotide sequence alignment of Cap genes between PCV2-GX-6 strain and other isolates. Base deletion site of PCV2-GX-6 was marked with red box. Base mutation site of PCV2-GX-6 was marked with green box. (**B**) Amino acid sequence alignment of Cap genes between PCV2-GX-6 strain and other isolates, mutation sites were marked with blue box. (**C**) Structure of PCV2-GX-6 Cap protein was predicted by Swiss-model (https://swissmodel.expasy.org/interactive), mapped, and compared with PCV2-JS17-8 (accession no.: MH211363) via Pymol.

Previous studies reported the PCV2d was the main prevalent genotype at present in China [7, 20–24]. In 2022, we successfully isolated and identified 5 PCV2 strains. Cap protein is the only structural protein of PCV2, which is the most important antigen to induce the production of specific antibodies and neutralizing antibodies in the host [25]. Therefore, the analysis of the mutation of the Cap gene of PCV2 is conducive to the further study of its epidemiological characteristics and antiviral reaction mechanism with virus escape from the host. DNAStar and Lasergene Megalign 7.1.0 were used to compare the ORF2 encoding genes of the 35 PCV2 reference strains to analyze the genetic evolution of isolates. The results showed that the homology with nucleotide and amino acid between the isolates and reference strains was 83.8-99.9% and 82.1-97.9%, respectively. The nucleotide and amino acid homology among the isolates was 99.7-100% and 97.4-100%, respectively. Phylogenetic analysis of ORF2 sequences of PCV2 was performed based on Lasergene Megalign 7.1.0 software, phylogenetic tree was constructed using the neighbor-joining method, with 1000 bootstrap replicates. According to the above results, all isolates belong to PCV2d genotype (Fig. 2). Among them, PCV2-GX-6 had the highest homology with the reference strain PCV2-JS17-8 (accession no.: MH211363). Compared with the reference strains, PCV2-GX-6 had a mutation, which was C200G, and the deletion of the base at site 30 (Fig. 3A). Further analysis of amino acid differences revealed the Cap protein of PCV2-GX-6 had a total of 6 mutations named R169T, D228T, P230H, N232T, P233L, K234S (Fig. 3B). No abrupt changes were found in T cell epitope region. Only one glycosylation site (143–145 amino acids) was relatively conserved, all of which were NYS. According to preliminary analysis, the mutation of amino acid may affect the antigenicity of PCV2-GX-6. PCV2-GX-6 Cap protein structure was predicted by Swiss-model (https://swissmodel.expasy.org/interactive), mapped, and compared with PCV2-JS17-8 (accession no.: MH211363) via Pymol (Fig. 3C). However, the structure of Cap protein between the PCV2-GX-6 and PCV2-JS17-8 showed the RMSD was 0.006Å, so there was no significant difference with each other.

### Pathogenicity in mice

**Fig. 4:**
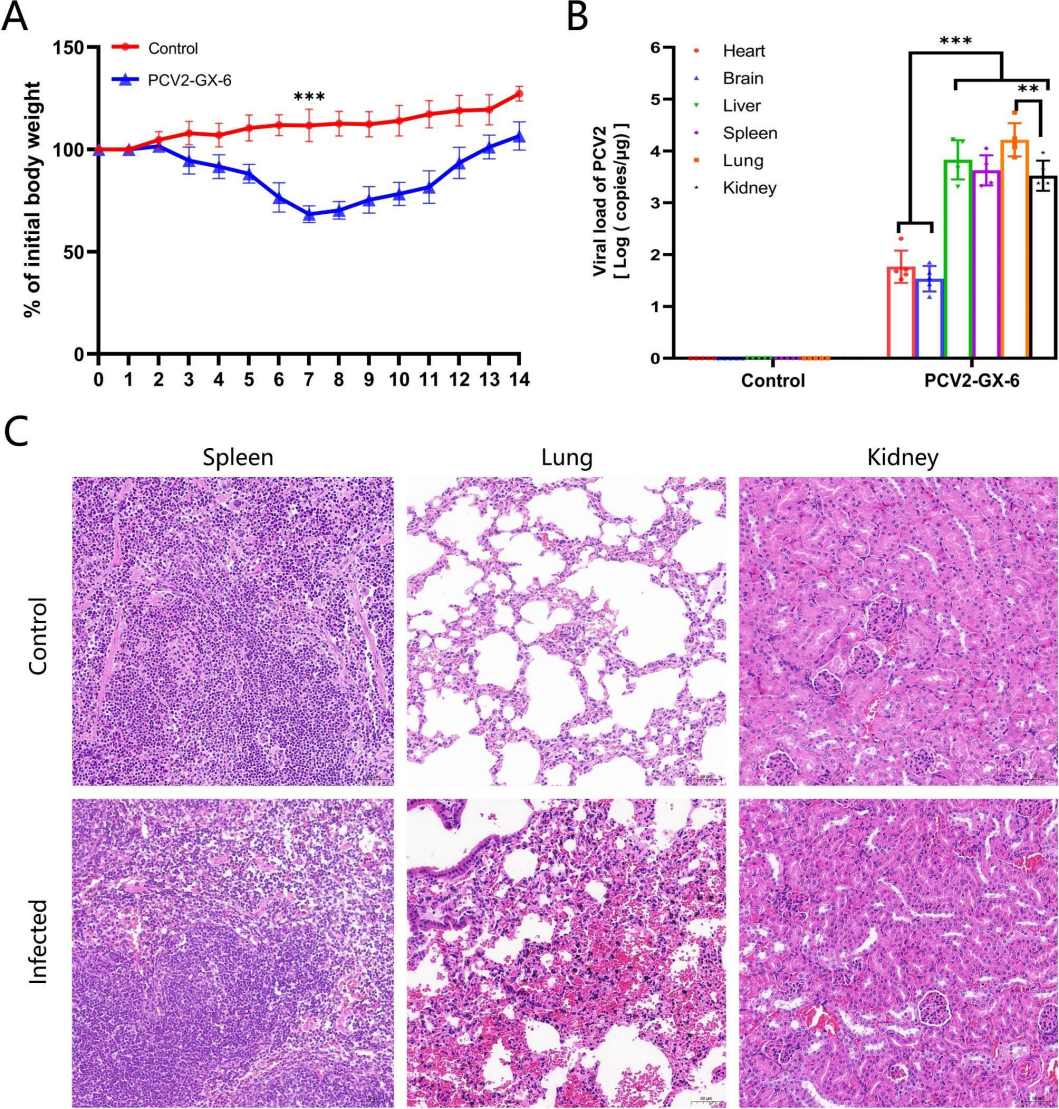
Pathogenicity of the isolates in mice. (**A**) Weight change of mice during the 14 dpi. (**B**) Viral load of PCV2 in various tissues of infected mice. Various tissues (heart, liver, spleen, lung, kidney, brain) from infected mice were tested to determine the copy number of PCV2 by absolute qPCR. (**C**) Histopathological examination of spleen, lung, and kidney tissues. Representative images of hematoxylin-and-eosin staining from mice infected with PCV2-GX-6. Data are represented as the mean ± SD. *, p < 0.05. **, p < 0.01. ***, p < 0.001 (ANOVA).

Previous studies have shown that PCV2 induced significant pathological changes in infected BALB/c mice [26]. In our study, the mice were infected with 1 × 10^6^ TCID_50_ of PCV2-GX-4, PCV2-GX-6, PCV2-GX-11 and the control mice were infected with DMEM. At 7 dpi, there was no significant difference in body weight between infected mice with PCV2-GX-4, PCV2-GX-11 and control group. Notably, the weight loss in mice infected with PCV2-GX-6 was significant (Additional fle 2: Fig. S2). Therefore, we chosed PCV2-GX-6 for pathogenicity study. The mice were infected with 1 × 10^6^ TCID_50_ of PCV2-GX-6, and the negative control was infected with DMEM. Body weight of the mice was monitored for 14 days. The hearts, livers, spleens, lungs, kidneys, and brains of mice infected with PCV2-GX-6 were collected for virus copy number determination when significant clinical symptoms appeared. Infected mice showed obvious anorexia and weight loss from 3 dpi. At 7 dpi, mice infected with PCV2-GX-6 were significant emaciation and weight loss (about 43.4%) compared with control mice (p < 0.01) (Fig. 4A). The negative control group showed no obvious clinical symptoms, and the body weight increased by 11.76%. After 7 days, the weight of infected mice began to increase. The highest copy number of the virus in the lung at 7dpi, followed by effective multiplication in the liver, spleen, and lung (Fig. 4B). PCV2 was not be detected in tissues of control mice. These data suggested that PCV2-GX-6 is significantly pathogenic in mice.

### Histopathological to spleen, lungs, and kidney

Through histopathological observation, the pathogenicity of PCV2-GX-6 in mice could be more accurately determined. The spleen, lung, and kidney of infected mice showed obvious histopathological injuries. As shown in Fig. 4C, the spleen was mildly abnormal and apoptosis of somatic germinal lymphocytes, but no obvious edema and inflammatory cell infiltration were observed. The structure of the lungs was severely abnormal, the alveolar arrangement was disordered and the boundary was blurred. Meanwhile, the septum of alveolar thickened, and part of the alveolar atrophy and collapsed. Furthermore, there is bleeding in the alveolar, which were filled with red blood cells. The lungs showed diffuse infiltration of inflammatory cells. The kidney structure was moderately abnormal, the glomerular structure in the visual field was abnormal, some glomeruli atrophy, the number of mesangial cells in the glomeruli was reduced, capillary loops were lost, and some glomeruli were clearly lobed.

## Discussions

PCV2 is the main pathogen causing PCVAD [2]. PCV2 infections can cause a variety of clinical symptoms and manifestations, including a series of porcine circovirus diseases. Porcine circovirus type 2 was reported in China in 2000, and PCV2 was first detected and isolated from piglets with multiple system failure syndrome in Beijing City and Hebei province. Previous studies have shown that PCV2 has a high mutation rate, which contributes to the polymorphism of the virus [24]. There are 9 genotypes of PCV2 (PCV2a, PCV2b, PCV2c, PCV2d, PCV2e, PCV2f, PCV2g, PCV2h and PCV2i) [3, 4]. At present, PCV2d is the main genotype, which is widely existing all over the world, and PCV2d can be subdivided into 2d-1 and 2d-2 [7, 8, 20]. In our study, PCV2-GX-4, PCV2-GX-6, PCV2-GX-7 PCV2-GX-11 and PCV2-GX-16 were detected from piglets in Guangxi. Homology and phylogenetic analysis indicated that isolates belonged to PCV2d genotype. PCV2-GX-6 was successfully isolated from PK-15 cells, which were stably cultivated for more than 33 passages. The results of PCR, IFA, WB, and TEM showed that PCV2-GX-6 was successfully isolated, the registration number was OQ800314. ORF2 sequence of PCV2-GX-6 had a base deletion at position 30 and a single mutation, which was C200G. Comparison with 35 reference strains showed that the nucleotide homology was highly consistent with MH211363.

Cap protein was the only structural protein of PCV2, which was the most important antigen to induce the production of neutralizing antibodies [25]. The specific sites of PCV2d genotype Cap were 53I, 59 K, 68 N, 89 L, 90T, 134 N, and 169R. Most of these amino acids were distributed along the surface of the viral Cap. It has been found that if one amino acid was mutated, the mutant virus will not be recognized by the epitope S antibody [27]. However, the base deletion of PCV2-GX-6 Cap did not affect the normal translation of 234 amino acids, but the R169T mutation occurred in 7 special amino acids of Cap protein. The structure analysis of Cap protein showed that the mutation of D228T, P230H, N232T, P233L, and K234S did not affect the structure of PCV2-GX-6. Therefore, the mutation of the Cap gene of PCV2-GX-6 was conducive to further study of its genetic evolution characteristics and antiviral reaction mechanism with virus escape from the host.

So far, PCV can be divided into 4 serotypes, including PCV1, PCV2, PCV3, and PCV4. Previous studies have shown that PCV1 has no obvious pathogenicity in pigs [28]. PCV3 and PCV4 can cause PDNS in pigs, reproductive disturbance in sows, multisystem inflammation, respiratory system disease, and abdominal diarrhea, but the clinical symptoms of PCV3 and PCV4 are not typical, and the pathogenesis needed to be further studied [15, 29]. At present, PCV2 is the most important pathogen responsible for post-weaning multisystem depletion syndrome (PMWS), porcine dermatitis, nephrotic syndrome (PDNS), porcine respiratory disease syndrome (PRDC), proliferative and necrotizing pneumonia (PNP), acute edema and respiratory diseases [30–34]. As well as, previous studies demonstrated that PCV2 was often co-infected with Porcine Reproductive and Respiratory Syndrome Virus (PRRSV), porcine parvovirus (PPV), Classical Swine Fever Virus (CSFV), and Porcine Pseudorabies Virus (PRV) [5, 35–38].

With the pathogenesis of PCV2 revealed. Previous research has reported the expression level of Cap determined the replication level of progeny viruses. After PCV2 infects cells, it promotes the expression of Cap and the replication of the viral genome by activating JNK, p38 MAPK, ERK, and PI3K/Akt signaling pathways [39–41]. Cap protein included major viral epitopes and plays an important role in the initiation of PCV2 replication. Cap protein interacted with six proteins in the cell, including MKRN1, p32, Par-4, NAP1, NPM1, and Hsp40. PCV2 strains mutated with Cap had significantly increased replication capacity and were highly pathogenic, and the clinical symptoms associated with PCV2 infection are more typical when PCV2b replaces PCV2a [42]. Therefore, it is necessary to study the pathogenicity of mutant strain PCV2-GX-6. Kiupel M et al. found that PCV2 can infect BALB/c mice and produce significant micropathological changes. The histopathological examination showed the proliferation, apoptosis, and necrosis of large lymphocytes in immune organs [26]. Li J et al. found that PCV2 successfully infected KM mice, which caused the lymph nodes and spleen histological lesions of lymph organ cell apoptosis and attenuation, pulmonary capillaries thrombosis interstitial pneumonia, and lymphocyte infiltration in liver tissue, blood vessels, there were inflammatory cells were stranded [43]. In our study, PCV2-GX-6 showed effective replication in vital organs of BALB/c mice, especially in the liver, spleen, lung, and kidney (Fig. 4B). These results found that the PCV2-GX-6 could be systemic spread in mice. Histopathological examinations showed that the virus could cause severe damage to respiratory tracts and proliferate effectively lungs (Fig. 4C). In the early stages of infection, respiratory symptoms and weight loss were significant in mice infected with PCV2-GX-6. The above results indicate that PCV2-GX-6 was highly pathogenic in mice.

Since the first report of PCV2 in China in 2000, with the transformation of dominant genotypes, PCV2 has been widely present in pig farms. Huang et al. found that the positive rate of PCV2 was 53% (3619/6872) in China from 2018 to 2020, and genetic evolution analysis found that PCV2d accounted for 79% (49/62 samples) [44]. However, the widespread epidemic of PCV2 was not curbed by the widespread use of vaccines. Due to the large genetic differences among different genotypes of PCV2, the matching degree between the vaccine and circulating strains is crucial for the prevention effect of PCV2 [45]. Therefore, it is necessary to monitor the virology of PCV2 and pay attention to the dominant genotypes prevalent in China, and the development of new vaccines containing multiple genotypes is of fundamental significance for the prevention and control of PCV2.

Interestingly, in mouse infection experiments, PCV2-GX-6 was more pathogenic than other isolates. Analysis of homology results between isolates revealed that PCV2-GX-6 had a base deletion (Fig. 3A), resulting in a high frequency of amino acid mutations. Whether the mutation of Cap amino acid sequence is conducive to the isolation and virulence enhancement of PCV2-GX-6 remains to be verified in the next step.

## Conclusion

PCV2-GX-4, PCV2-GX-6, PCV2-GX-7, PCV2-GX-11 and PCV2-GX-16 were isolated from piglets with clinical emaciation and respiratory diseases from Guangxi Province in China. PCV2-GX-6 was stably cultured in PK-15 cells for more than 30 passages. Homology and phylogenetic analysis showed that the isolates belonged to the PCV2d genotype. PCV2-GX-6 was closely related to the reference strain PCV2-JS17-8 (Genebank accession no.: MH211363). PCV2-GX-6 had a total of 6 amino acid mutations (R169T, D228T, P230H, N232T, P233L, K234S), but the mutations did not affect protein structure. The pathogenicity of mice showed that PCV2-GX-6 had a significant inhibitory effect on body weight, and could cause typical lesions of spleen, lung, and kidney in mice.

### Electronic supplementary material

Below is the link to the electronic supplementary material.

**Additional file 1**. Table S1: Primers used for co-infection detection in this study.

**Additional file 1**. Table S2: Primers used for PCV2 genome amplification in this study.

**Additional file 2**. Fig. S1: IFA results of PCV2-GX-4, PCV2-GX-7, PCV2-GX-11 and PCV2-GX-16 in PK-15 cells.

**Additional file 2**. Fig. S2: The result of body weight in mice infected with PCV2-GX-4, PCV2-GX-6, PCV2-GX-11 and DMEM.

**Additional file 2**. Fig. S3: Nucleotide sequence alignment of whole genome between PCV2-GX-6 strain and other isolates. Base deletion site of PCV2-GX-6 was marked with red box.

**Supplementary Material 3**

## Data Availability

The data that support the findings of this study are available from the corresponding author upon reasonable request.

## Abbreviations

PCV2: Porcine circovirus type 2
ORF: Open reading frame
PCR: Polymerase chain reaction
RT‑PCR: Reverse‑transcription polymerase chain reaction
qPCR: Quantitative polymerase chain reaction
M: Matrix protein of IAV
TCID_50_: 50% tissue culture infective dose
IFA: Indirect immunofluorescence assay
WB: Western blot assay
TEM: Transmission electron microscopic
SDS-PAGE: Sodium dodecyl sulfate polyacrylamide gel electrophoresis
HE: Hematoxylin-and-eosin
dpi: Days post-infection
CSFV: Classical swine fever virus
PRRSV: Porcine reproductive and respiratory syndrome virus
PRV: Porcine pseudorabies virus
PCV3: Porcine circovirus type 3
PCV4: Porcine circovirus type 4
