# Supplementary material for: Isolation and pathogenicity of porcine circovirus type 2 in mice from Guangxi province, China
Source: Virol J. 2023 Aug 29;20:195. doi: 10.1186/s12985-023-02161-5 (PMC10466715; doi:10.1186/s12985-023-02161-5)
Supplement: Supplementary file 1 — Additional file 1. Table S1: Primers used for co-infection detection in this study. [file 12985_2023_2161_MOESM1_ESM.docx]

**Table S1** Primers used for co-infection detection in this study

| **Primers** | **Sequence (5’ to 3’)** | **Length (bp)** | **Target** |
| --- | --- | --- | --- |
| CSFV-F | TAGGGTGGACGGGTGTCATAGAGT | 536 | E2 |
| CSFV-R | AAGCATATATTGCTGGAAGTAGCT |  |  |
| PRRSV-F | TCCCAGTGCCAAGTGGACAT | 500 | GP2 |
| PRRSV-R | CAGCGGAAACCAAAAACAGTAC |  |  |
| PRV-F | GCTGACGCTGACGACGGTCCCCT | 491 | gD |
| PRV-R | CGCCGAACTTGTACGTGCGGTGCT |  |  |
| PCV3-F | AGGGAAAGCCCGAAACACAG | 395 | replication-associated protein |
| PCV3-R | GCAGGCATCTTCTCCGCAAC |  |  |
| PCV4-F | TCGAGCGGCGCGAGATTG | 426 | replication-associated protein |
| PCV4-R | CCACTTCACTCATTGAGCTGCCG |  |  |
