# Supplementary material for: Isolation and pathogenicity of porcine circovirus type 2 in mice from Guangxi province, China
Source: Virol J. 2023 Aug 29;20:195. doi: 10.1186/s12985-023-02161-5 (PMC10466715; doi:10.1186/s12985-023-02161-5)
Supplement: Supplementary file 2 — Additional file 1. Table S2: Primers used for PCV2 genome amplification in this study. [file 12985_2023_2161_MOESM2_ESM.docx]

**Table S2** Primers used for PCV2 genome amplification in this study

| **Primers** | **Sequence (5’ to 3’)** |
| --- | --- |
| PCV2-F | GAACCGCGGGCTGGCTGAACTTTTGAAAGT |
| PCV2-R | GCACCGCGGAAATTTCTGACAAACGTTACA |
