# Supplementary figures and images for: Isolation and pathogenicity of porcine circovirus type 2 in mice from Guangxi province, China
Source: Virol J. 2023 Aug 29;20:195. doi: 10.1186/s12985-023-02161-5 (PMC10466715; doi:10.1186/s12985-023-02161-5)

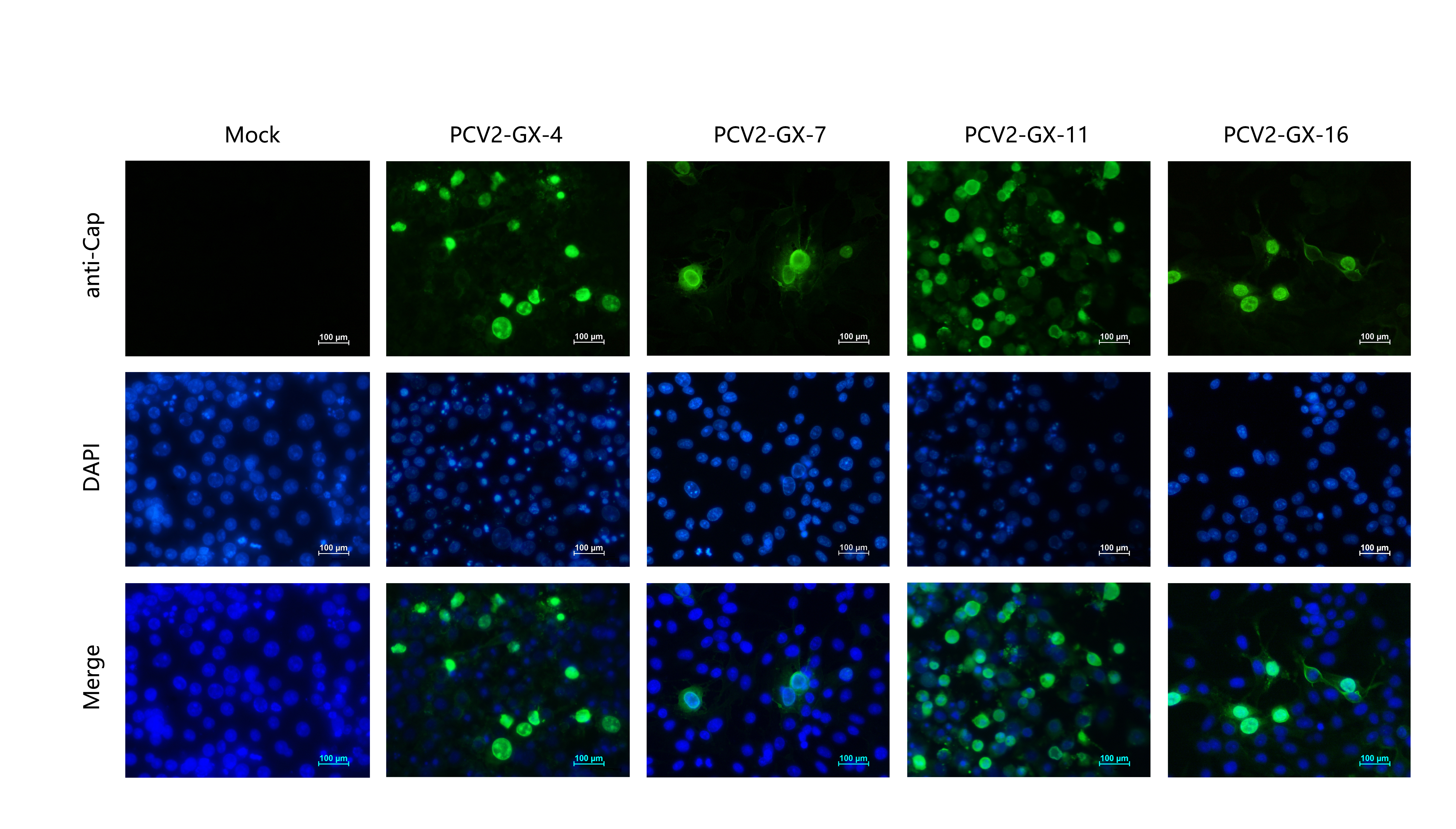

Supplement: Supplementary file 3 — Additional file 2. Fig. S1: IFA results of PCV2-GX-4, PCV2-GX-7, PCV2-GX-11 and PCV2-GX-16 in PK-15 cells. [file 12985_2023_2161_MOESM3_ESM.jpg]

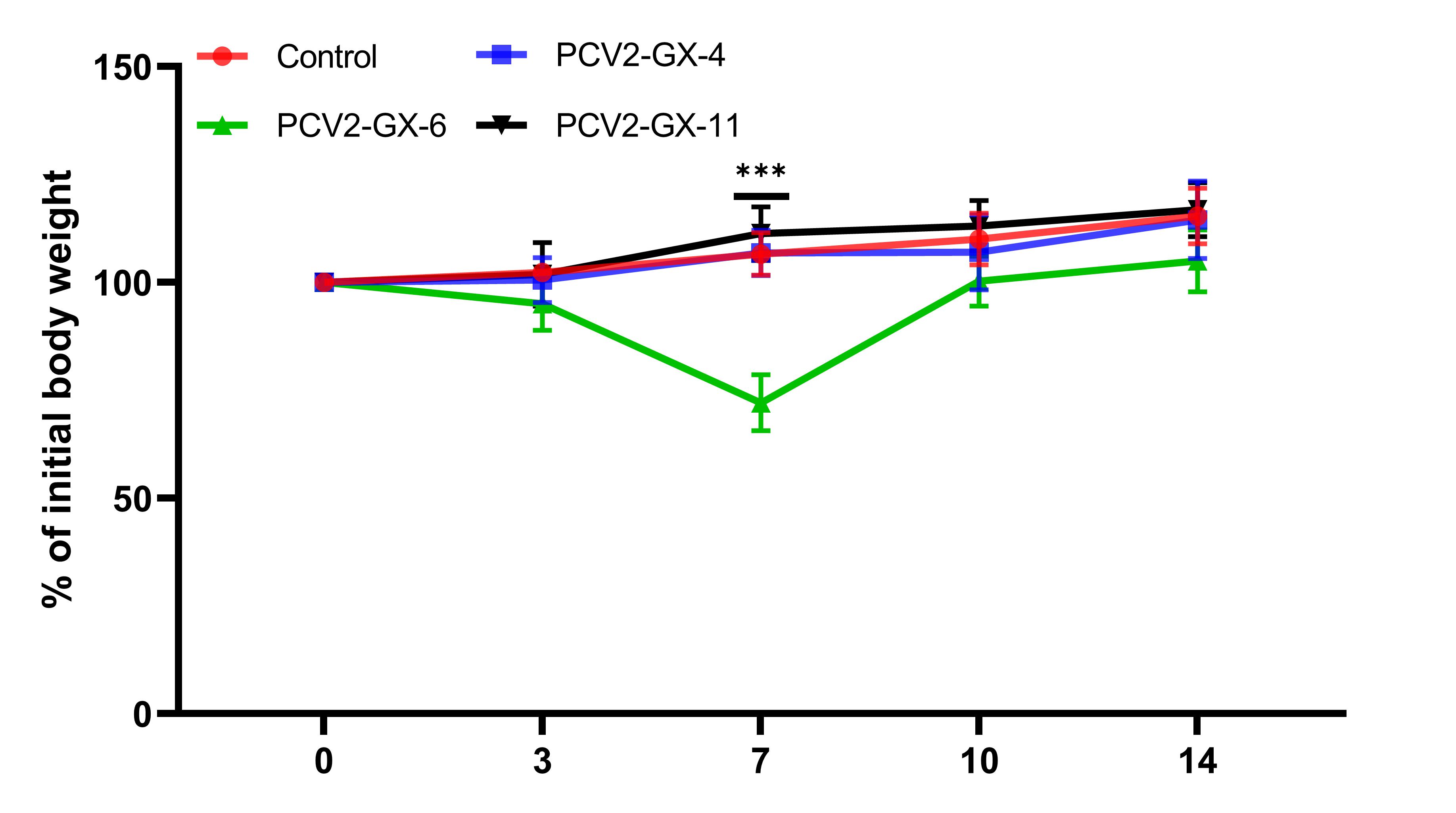

Supplement: Supplementary file 4 — Additional file 2. Fig. S2: The result of body weight in mice infected with PCV2-GX-4, PCV2-GX-6, PCV2-GX-11 and DMEM. [file 12985_2023_2161_MOESM4_ESM.jpg]

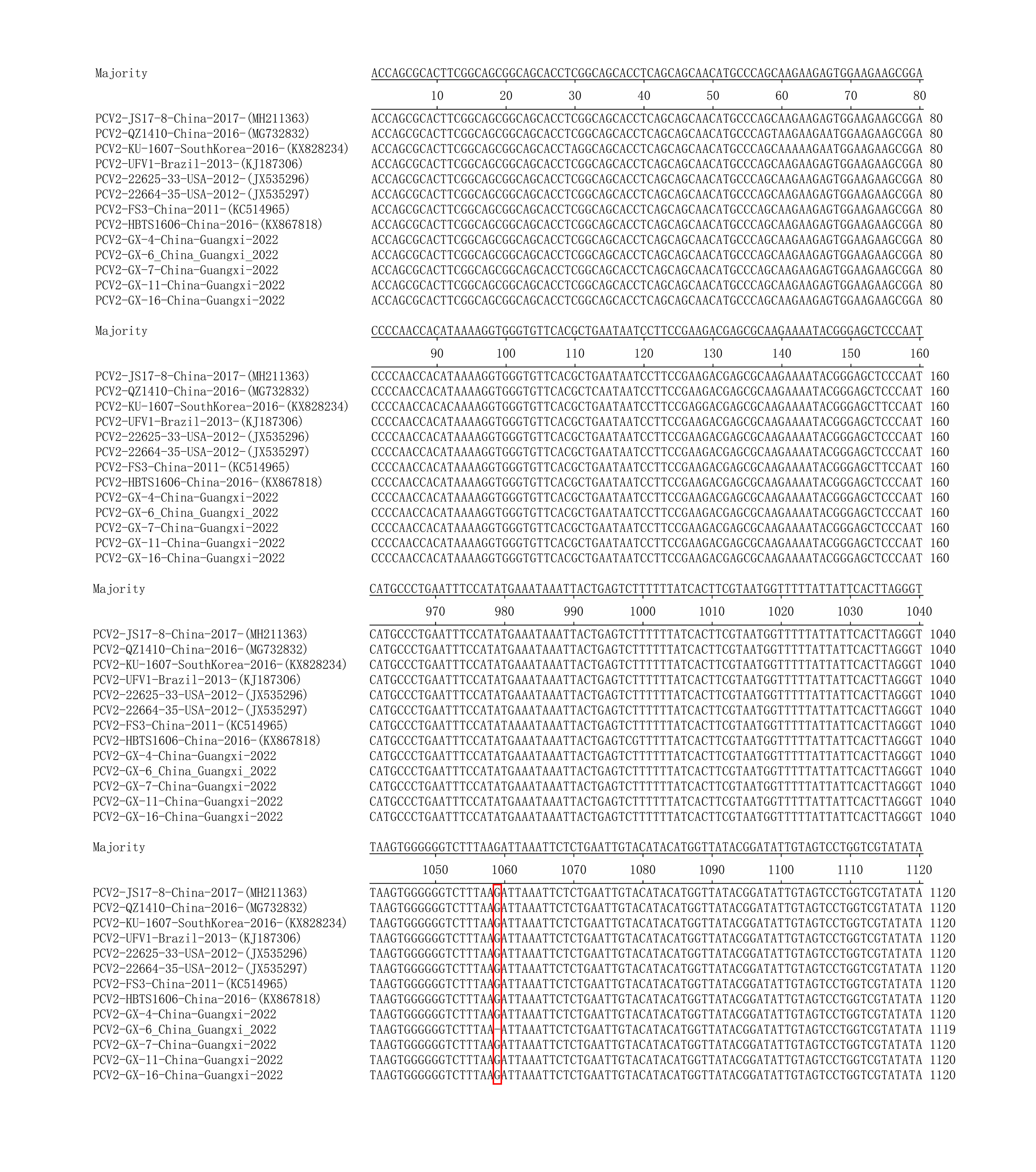

Supplement: Supplementary file 5 — Additional file 2. Fig. S3: Nucleotide sequence alignment of whole genome between PCV2-GX-6 strain and other isolates. Base deletion site of PCV2-GX-6 was marked with red box. [file 12985_2023_2161_MOESM5_ESM.jpg]

**Raw data Fig.1A**


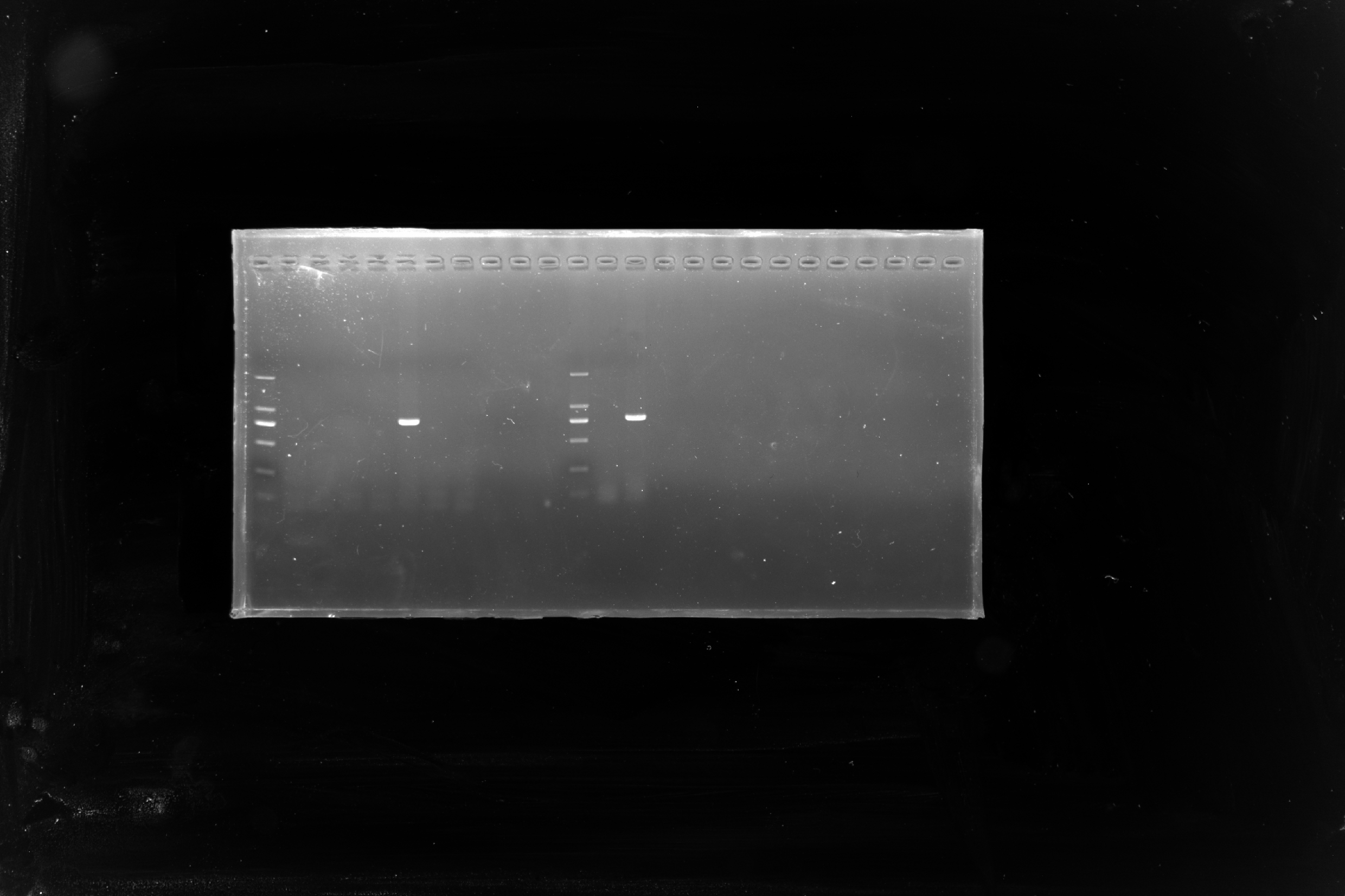


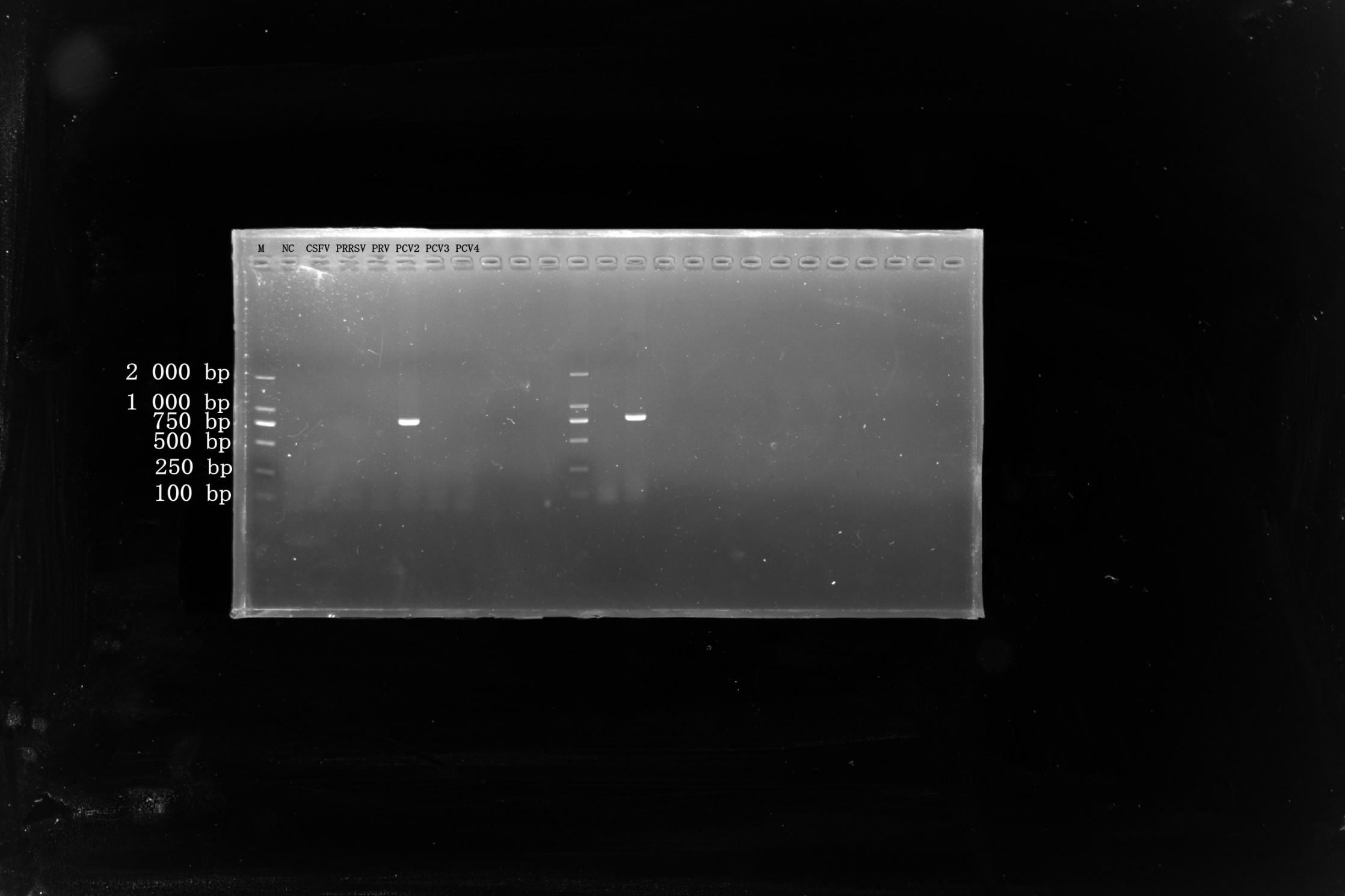


**Raw data Fig.1C**


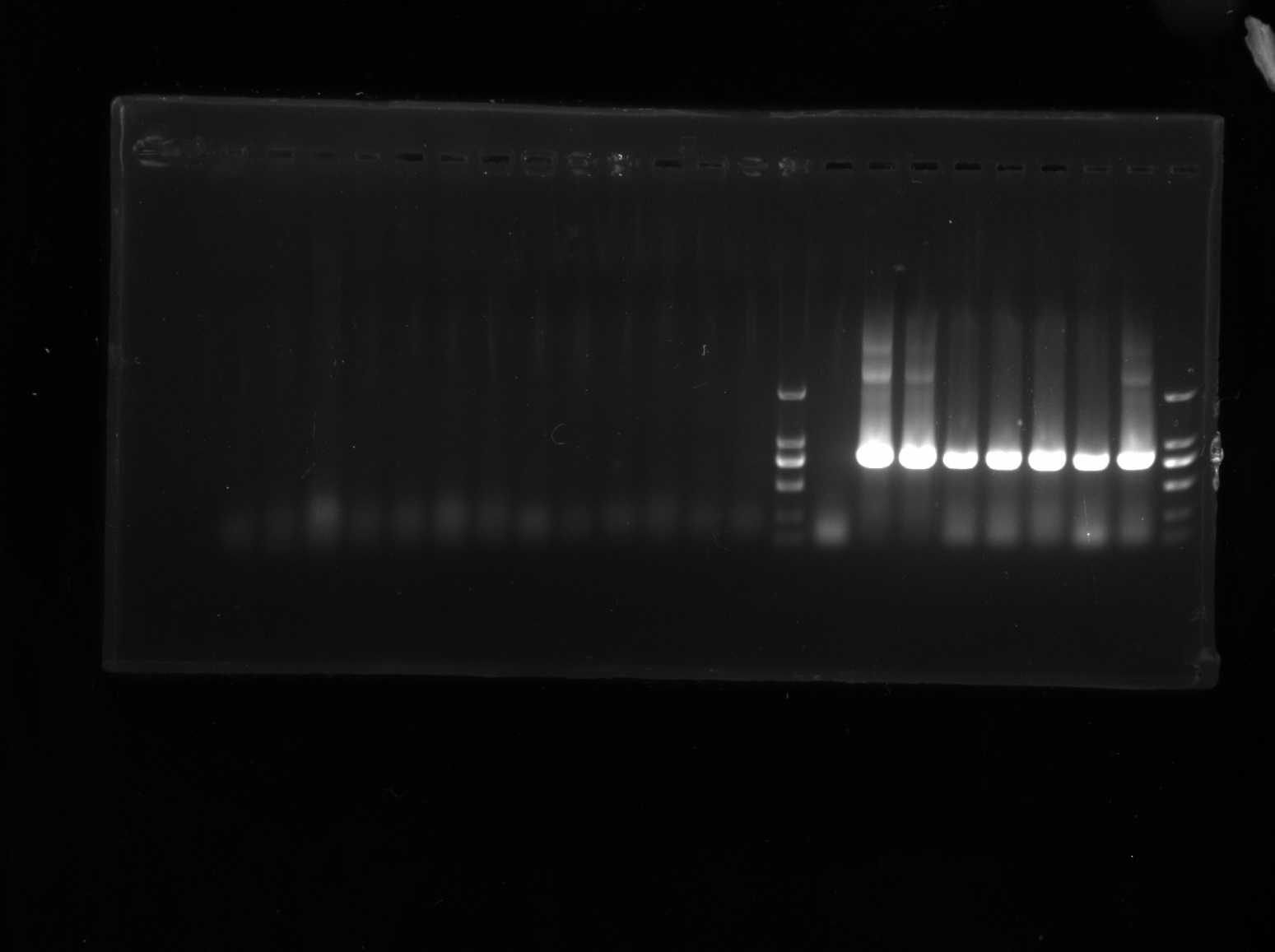

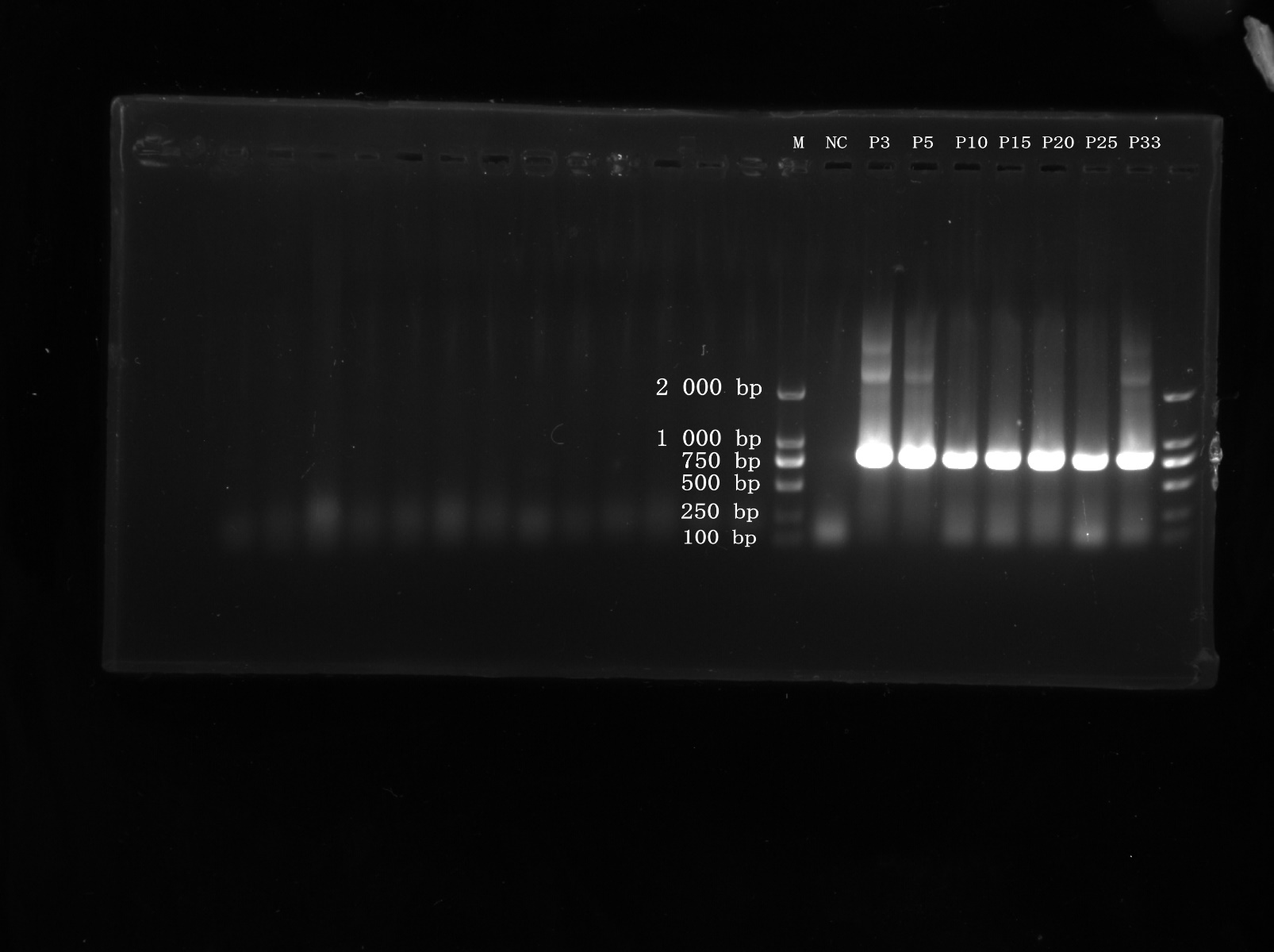


**Raw data Fig.1D**


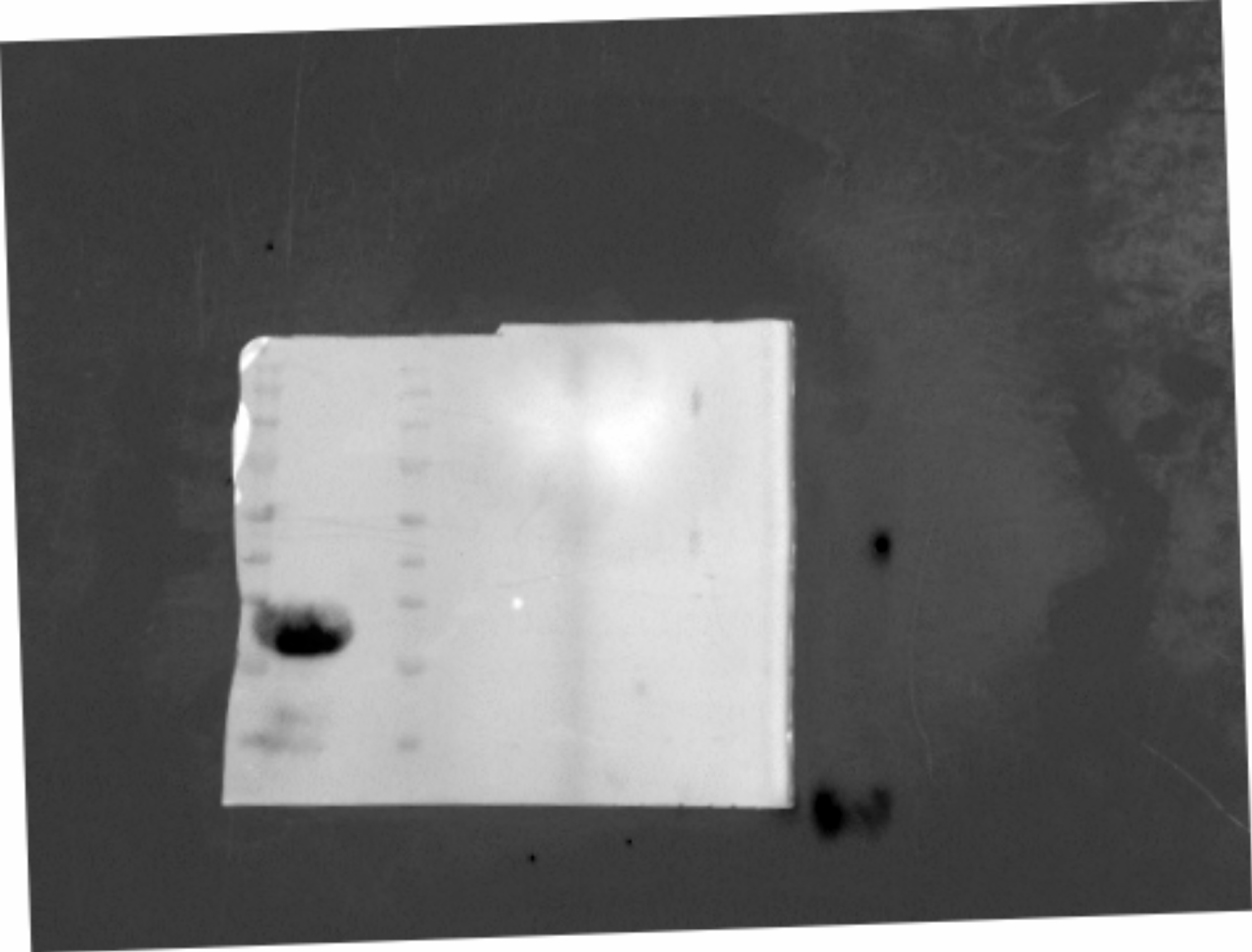

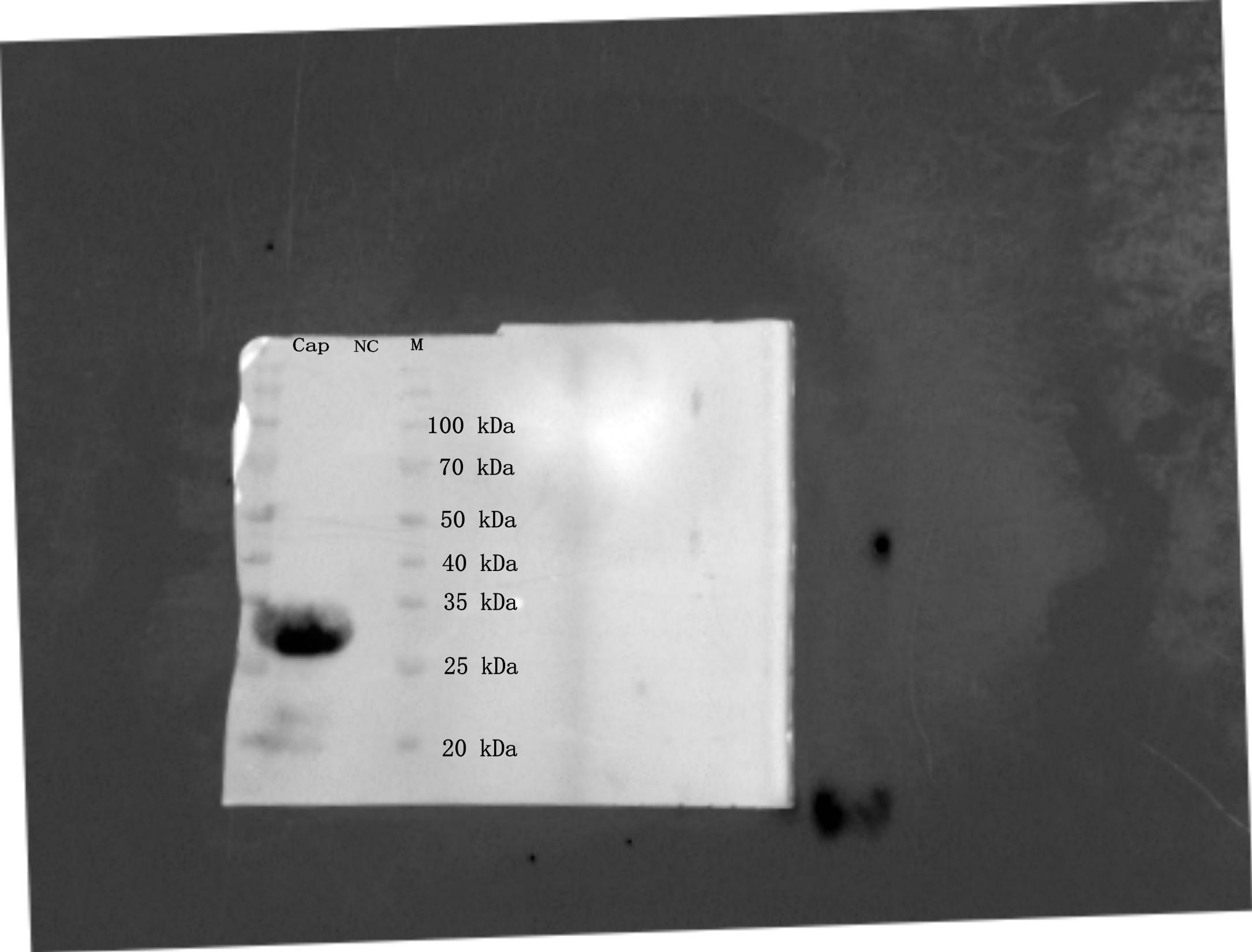

Supplement: Supplementary file 6 — Supplementary Material 3 [file 12985_2023_2161_MOESM6_ESM.docx]
